# Web GIS in practice VI: a demo playlist of geo-mashups for public health neogeographers

**DOI:** 10.1186/1476-072X-7-38

**Published:** 2008-07-18

**Authors:** Maged N Kamel Boulos, Matthew Scotch, Kei-Hoi Cheung, David Burden

**Affiliations:** 1 Faculty of Health and Social Work, University of Plymouth, Drake Circus, Plymouth, Devon, PL4 8AA, UK; 2 Center for Medical Informatics, School of Medicine, Yale University, New Haven, CT, USA; 3 Departments of Anesthesiology and Genetics, School of Medicine, and Department of Computer Science, Yale University, New Haven, CT, USA; 4 Daden Limited, 103 Oxford Rd, Moseley, Birmingham, B13 9SG, UK

## Abstract

'Mashup' was originally used to describe the mixing together of musical tracks to create a new piece of music. The term now refers to Web sites or services that weave data from different sources into a new data source or service. Using a musical metaphor that builds on the origin of the word 'mashup', this paper presents a demonstration "playlist" of four geo-mashup vignettes that make use of a range of Web 2.0, Semantic Web, and 3-D Internet methods, with outputs/end-user interfaces spanning the flat Web (two-dimensional – 2-D maps), a three-dimensional – 3-D mirror world (Google Earth) and a 3-D virtual world (Second Life ^®^). The four geo-mashup "songs" in this "playlist" are: 'Web 2.0 and GIS (Geographic Information Systems) for infectious disease surveillance', 'Web 2.0 and GIS for molecular epidemiology', 'Semantic Web for GIS mashup', and 'From Yahoo! Pipes to 3-D, avatar-inhabited geo-mashups'. It is hoped that this showcase of examples and ideas, and the pointers we are providing to the many online tools that are freely available today for creating, sharing and reusing geo-mashups with minimal or no coding, will ultimately spark the imagination of many public health practitioners and stimulate them to start exploring the use of these methods and tools in their day-to-day practice. The paper also discusses how today's Web is rapidly evolving into a much more intensely immersive, mixed-reality and ubiquitous socio-experiential Metaverse that is heavily interconnected through various kinds of user-created mashups.

## Background

GIS (Geographic Information Systems and Science) have always shared many of the foundational ethea (plural of ethos) of Web 2.0 [1], (even before the latter became known as a distinct entity), namely data sharing, remixing and repurposing, and collaboration. GIS enable remixing and repurposing of data by "mashing-up" various data and map layers or themes from multiple sources into one study/map (with multiple layers covering same locations superimposed like onion's skin). And now with the advent of Web 2.0 technologies, the democratization and participatory nature of GIS have never been more possible or powerful. Neogeography and the GeoWeb 2.0 have been born and unleashed for use by the masses [2,3]!

### On mashups and their growing popularity and importance

'Mashup' was originally used to describe the mixing together of musical tracks to create a new piece of music [4]. The term now refers to Web sites or services that weave data from different sources into a new data source or service. Mashups are becoming increasingly widespread, especially in the context of combining geographic data and displaying such integrated data on maps. Web-based mapping applications like Google Maps [5] and Google Earth [6] allow multiple independently generated datasets encoded using the Keyhole Markup Language (KML) format to be mixed and displayed via a two-dimensional – 2-D map (or three-dimentional – 3-D globe in the case of Google Earth) [7]. The latest offerings from Google Maps, namely My Maps [8] and Mapplets [9], have made it possible for anyone to create and share their own interactive online maps with just a few mouse clicks and no (or almost no) coding at all! (With Google Mapplets, anyone can tap into, remix and reuse third-party mini-applications for Google Maps (known as Mapplets) from a rapidly expanding catalogue maintained by Google, to create and share even more powerful personal maps.)

Many scientists have also utilized these technologies for research purposes [10]. For example, *Nature *has created its own geo-mashup using Google Earth for tracking avian-flu outbreaks [11], and HealthMap [12], developed by the Children's Hospital Informatics Program in Boston, brings together disparate data sources within Google Maps to achieve a unified and comprehensive view of the current global state of infectious diseases and their effect on human and animal health. This freely available Web site integrates outbreak data of varying reliability, ranging from news sources (e.g., Google News [13]) to curated personal accounts (e.g., ProMED [14]) to more valid alerts (e.g., World Health Organization [15]). Other public health mashup work can be browsed at [16]. These examples represent a class of Web-based neogeography applications that combine the complex techniques of cartography and Geographic Information Systems (GIS) and place them within reach of users [17]. The benefit of such easy-to-use GIS applications is evident in an increasing diversity and quantity of publicly available geocoded health data and a growing interest in using GIS and other Web-based tools for mashup of public health data.

It is therefore not surprising but rather commendable that the UK government has recently launched a data mashup competition to find innovative ways of using the masses of data it collects [18]. The government is hoping to find new uses for public information in the areas of criminal justice, health and education, and is opening up gigabytes of information for this purpose from a variety of sources like mapping information from Britain's Ordnance Survey, medical information from the NHS (National Health Service), and neighbourhood statistics from the Office for National Statistics. (None of the data is personal information.)

### How mashups work – the basic principles

Over the last few years, the complexity and magnitude of research data with advances in genomic sequencing and translational science have increased the need for complex mashup applications. One possible solution is Web 2.0, a term that describes the rising global trend in use of World Wide Web technology and Web design in the past few years, and represents applications that aim to enhance creativity, information sharing, and collaboration among users. Web 2.0 comprises online services that promote interaction between users and cooperative development of Web resources [1,19]. These technologies, tools, and sites can be broadly categorized as follows:

#### • Data formatter

The contents provided by different Web sites are organized and displayed in many different ways. The traditional approach to extracting Web content and reformatting it is to write specific screen-scraping programs to extract content from specific sites. This approach is not scalable given the high degree of heterogeneity involved. Also, it requires a significant amount of programming effort. To address this, tools such as Dapper [20] provide the user with the ability to visually map the Web content to a particular structure. In addition, these tools allow the extracted content to be output in different formats such as RSS (Really Simple Syndication – described in [2]). These tools ease the effort of content extraction and formatting over the Web [21].

#### • Data connector

To facilitate mashup of data provided by different sites in different formats, tools such as Yahoo! Pipes [22] have been developed to allow users to graphically create a Pipe or workflow to connect data including those generated by other tools like Dapper. Such tools can directly accept data in different formats and integrate them. The integrated data can be formatted in different ways for analysis purposes [21].

#### • Data visualization

Once multiple datasets are parsed or integrated in a common format, tools are available for visualizing data in an integrated fashion. For example, Yahoo! Pipes can be used to integrate and format geo-referenced data into the KML format for visualization by Google Maps or Google Earth [23].

#### • Data sharing

One important aspect of Web 2.0 is data sharing and community collaboration. For example, Dapper and Yahoo! Pipes both contain collaboration forums in which users can view and utilize the work of others. In the context of GIS, Web 2.0 sites such as GeoCommons [24] allow geo-referenced data (e.g., KML files) to be tagged, shared, reused, and remixed [21].

#### • Web API

Another key part of the Web 2.0 trend features the growing use of various Web Application Programming Interfaces (APIs) for developers to build rich client applications that can programmatically access online services such as Google Maps and GeoCommons. Such Web APIs allow existing functionalities to be reused. For example, using the GeoIQ JavaScript API provided by GeoCommons [25], one can develop client applications that include content such as heat maps, concentration indices, or intersection indices in custom data [21].

These Web 2.0 technologies, tools, and services, in conjunction with neogeography applications such as Google Maps and Google Earth, can support public health research, including infectious disease surveillance and molecular epidemiology. They reduce the onus of the public health expert to write complex programming code to perform data integration. They also promote data sharing and community collaboration. Whether the purpose is to analyze historical trends of data over time or to detect disease anomalies in real-time, Web 2.0 technology can easily integrate numerical and spatial data for public health decision support.

Using a musical metaphor that builds on the origin of the word 'mashup', this paper will present a demonstration "playlist" of four practical geo-mashup example and idea sets that make use of a range of Web 2.0, Semantic Web, and 3-D Internet methods, with outputs/end-user interfaces spanning the flat Web (2-D maps), a 3-D mirror world (Google Earth) and a 3-D virtual world (Second Life ^®^).

## A demo "playlist"

### Mashup song #1: Web 2.0 and GIS for infectious disease surveillance

**Figure 1:**
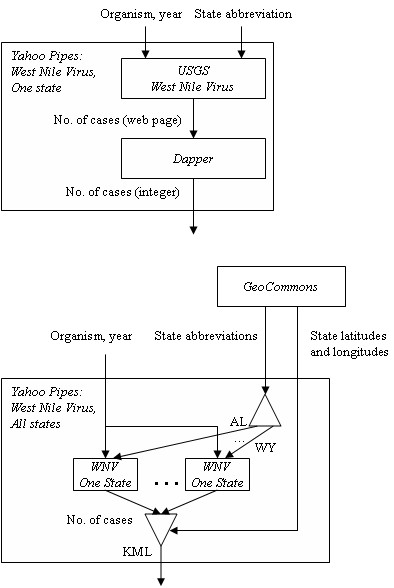
Web 2.0 for public health research.

Web 2.0 can be utilized along with GIS for infectious disease surveillance. Figure 1 shows a flow diagram for development of a Web 2.0 mashup application for West Nile Virus (WNV) surveillance. The first step involves the use of Dapper [20] to obtain WNV data from the United States Geological Survey (USGS) Web site [26]. The data contains the number of WNV cases per each state for animals (such as horses and birds), humans, and mosquitoes. The second step involves geo-referencing the data by using Yahoo! Pipes [22] to mashup the WNV case data with GeoCommons [24], a Web 2.0 mapping service.

**Figure 2:**
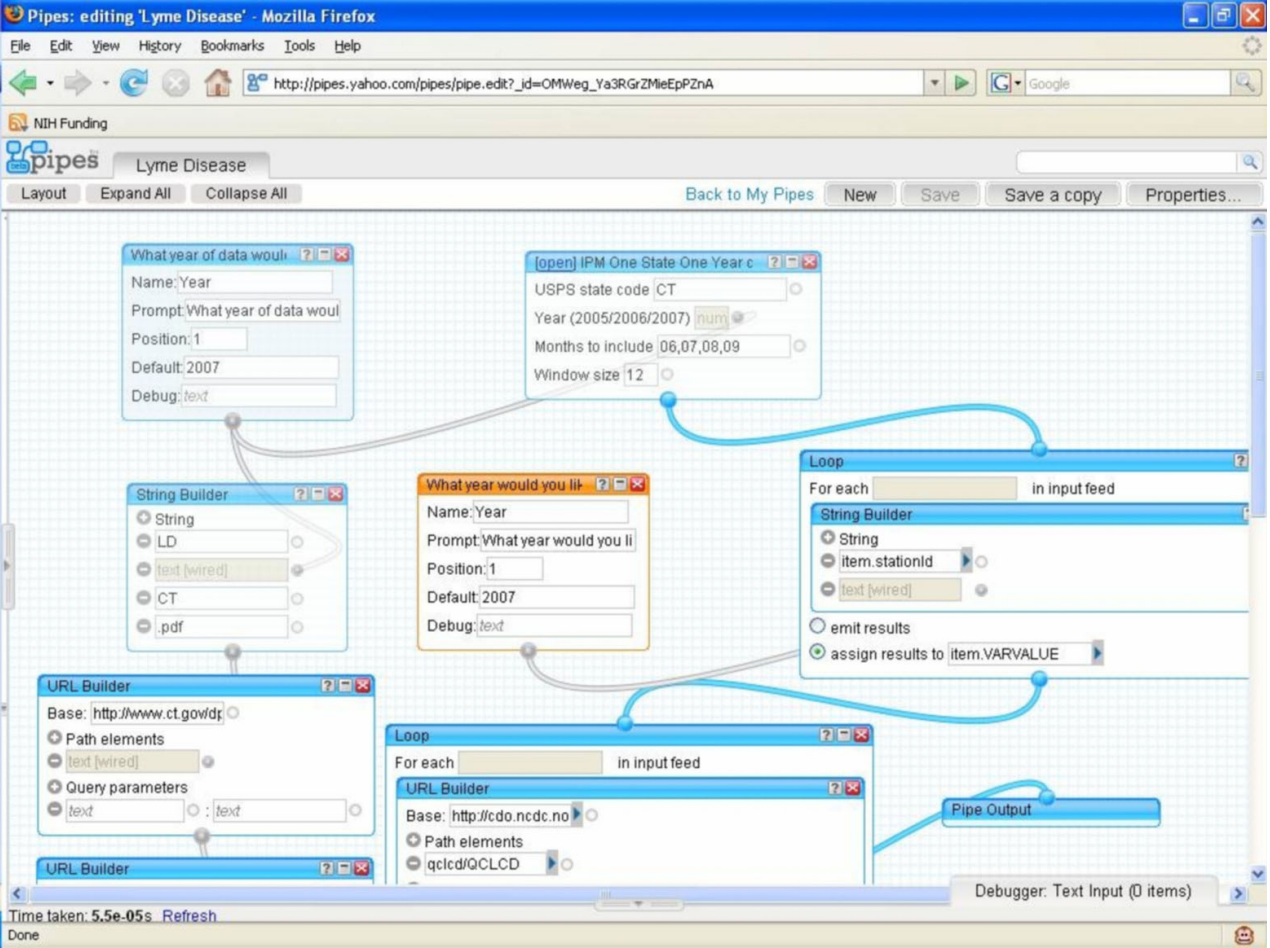
The mashup of WNV surveillance data in Yahoo! Pipes.

The ability to mashup and integrate Web-based surveillance data lies with the fact that Yahoo! Pipes contains functions to retrieve data streams, store them locally as a list, perform iterative loops, string functions, regular expressions, Web services, and geo-referencing. Figure 2 shows the corresponding Yahoo! Pipe for the mashup of WNV surveillance data.

**Figure 3:**
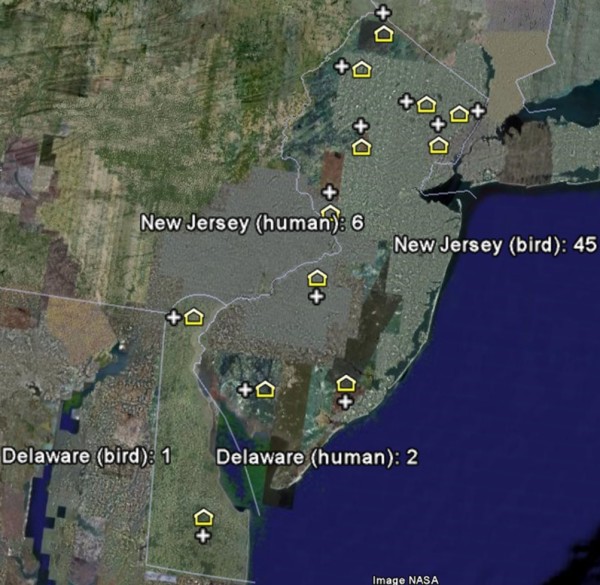
GIS display of Web 2.0 mashup for West Nile Virus surveillance using Google Earth.

Finally, a Web 2.0 mashup can support complex calculations that are often required in epidemiology and infectious disease surveillance. For example, temperature is a significant factor in the transmission of many vector-borne diseases. In our West Nile Virus example, higher temperatures enable for the Extrinsic Incubation Period (EIP) to be completed within the mosquito, which indicates that the virus reaches infectivity and can be transmitted to a susceptible host such as a human, bird, or horse [27]. Scientists need to track temperature data and calculate measures such as 'degree days' [28] to determine whether the temperature supports virus transmission. Degree days can be calculated using sine methods [28] and may be too difficult to compute using GIS. Web 2.0 supports the integration of Web resources to perform such calculations and combine the results with geo-referenced data for WNV surveillance. Our Yahoo! Pipe (Figure 2) fetches temperature data from the National Climate Data Center Web site [29]. The output of Yahoo! Pipes can be a KML file for display in Google Earth (Figure 3) or Google Maps. In this example, weather stations are shown with '+' to indicate that the 2005 summer temperature in the area supports the risk of West Nile Virus transmission. Numbers of positive bird and human WNV cases in 2005 are also shown.

### Mashup song #2: Web 2.0 and GIS for molecular epidemiology

**Figure 4:**
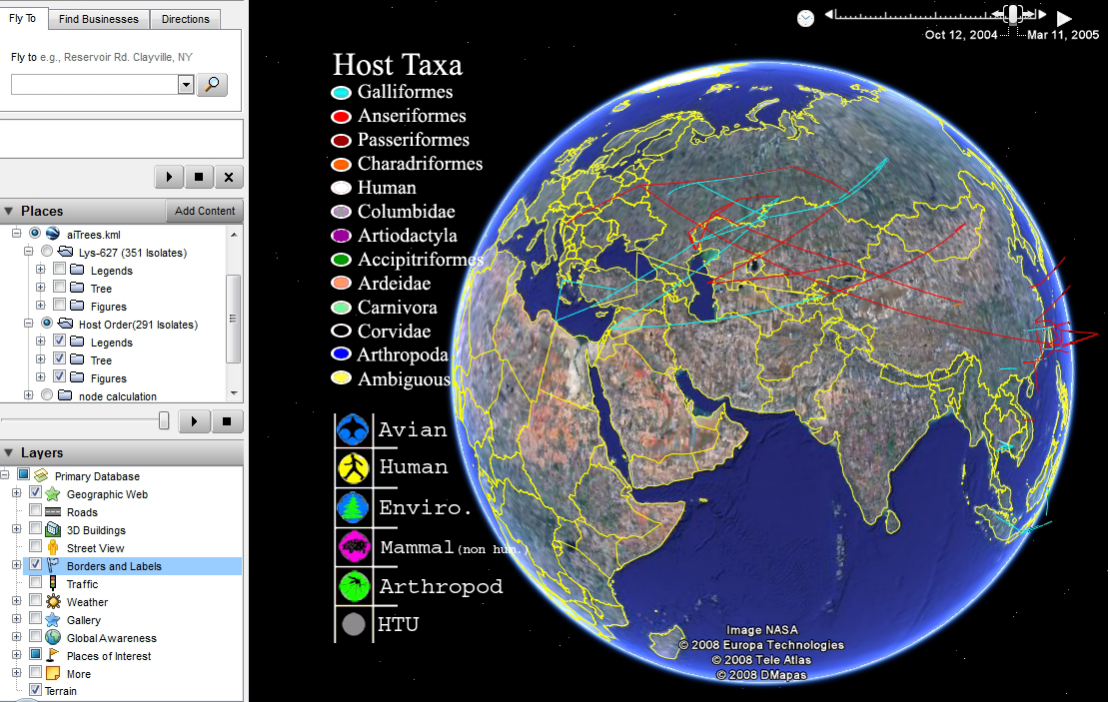
**Geographic visualization of the spread of avian influenza (H5N1) in Google Earth**. Screenshot of the freely available Google Earth file by Daniel Janies and colleagues, which they describe in their paper [31].

Web 2.0 can also support the mashup of molecular information in GIS. For example, phylogenetic analysis through the use of tree generation software has recently been integrated in Google Earth [30]. The project called TreeBASE II enables for scientists to analyze genetic distances across different isolates and subtypes, and harnesses Google Earth to deliver biological information with a geographic component [31] (Figure 4).

Another resource for mashup of molecular data is the Mesquite Project [32]. The modular system promotes collaboration among scientists to develop their own programs or modules and then upload the modules for other programmers to utilize and enhance. This mashup approach enables modules to be attached to other modules for creation of a hybrid module. There is great potential for GIS to be included as a module for Mesquite in as much the same way that TreeBase II presents trees within GIS.

Since our demo "playlist" was created based on the original concept of "musical mashup", it is also very possible for these separately-composed "songs" to be remixed; for example, different species of mosquitoes carrying the WNV (see "Mashup song #1" above) can be queried semantically (ontologically – see "Mashup song #3" below), studied using the geo-phylogenetic tree ("Mashup song #2"), and visualized/interacted with in an avatar-inhabited 3-D virtual world environment (see "Mashup song #4" below).

### Mashup song #3: Semantic Web for GIS mashup

Despite the emergence of Web 2.0 tools like Yahoo! Pipes and standard geo-data formats like KML, the task of identifying and integrating datasets of interest must be manually done by people. 'Semantic mashup' is a conception in which computers help humans discover and integrate data. A semantically-enriched machine readable format is needed for implementing the vision of semantic mashup. GeoRSS (an extension of RSS) is a step in this direction [2]. While a regular RSS feed is used to describe feeds (channels) of Web content such as news articles, Web content consisting of geographical elements such as latitudes and longitudes can be described using GeoRSS. Like RSS feeds that are consumed by feed readers and aggregators, GeoRSS feeds are designed to be consumed by geographic software such as map generators.

GeoRSS can be viewed as an application of RDF (Resource Description Framework), since RSS 1.0 is a language of RDF. RDF is part of a broader technology called 'Semantic Web' [33-35], which is a set of recommendations and specifications supported by the World Wide Web Consortium (W3C) [36]. The Semantic Web emphasizes common formats and languages for semantic interoperability. For example, RDF enables for the integration and combination of data drawn from diverse sources. This is an enhancement from the original Web which emphasized the interchange of documents. The Semantic Web also supports languages such as SPARQL (a recursive acronym that stands for SPARQL Protocol and RDF Query Language), which can be used to express queries across diverse data sources, whether the data are stored natively as RDF or viewed as RDF via middleware. SPARQL is much suited for recording how Web content relates to real world objects. This allows a Web reference, such as a person, or a machine, to start off in one database, and then move through an unending set of databases which are connected not by wires but by relationships.

The use of ontologies, or formal representations of concepts and their relationships, has been a popular method for supporting complex knowledge representation in the Semantic Web [34,35]. For example, an expressive ontology language called the Web Ontology Language (OWL) is now a W3C recommendation [37]. OWL-based ontologies can support sophisticated queries as well as machine reasoning and inferencing. The GeoNames Ontology [38] is an example of geo-ontology available in OWL format. It is part of GeoNames [39], which is a database integrating geographical data such as names of places in various languages, elevation, population and other features from various sources. The GeoNames Ontology makes it possible to add geospatial semantic information to the Web. The ontology distinguishes the 'Concept' from the 'Document'. For example, the town Embrun in France is associated with two URIs (Uniform Resource Identifiers): [40] and [41]. The first URI [40] identifies the town Embrun in France. The second URI [41] is the RDF document with the information GeoNames has about Embrun. The GeoNames Web server is configured to redirect requests for the first URI to the second URI. The redirection tells Semantic Web Agents that Embrun is not residing on the GeoNames server but that GeoNames has information about it instead.

The elements in the GeoNames ontology are semantically interlinked with each other in the following ways:

#### • Children

These include countries for a continent, subdivisions, etc. For example, the children of France include Auvergne (province) and Lorraine (administrative region).

#### • Neighbours

These are neighbouring countries for a given country. For example, Switzerland and Germany are neighbours of France.

#### • Nearby features

For example, nearby the Eiffel Tower are Champ de Mars and Trocadéro – Palais de Chaillot.

**Figure 5:**
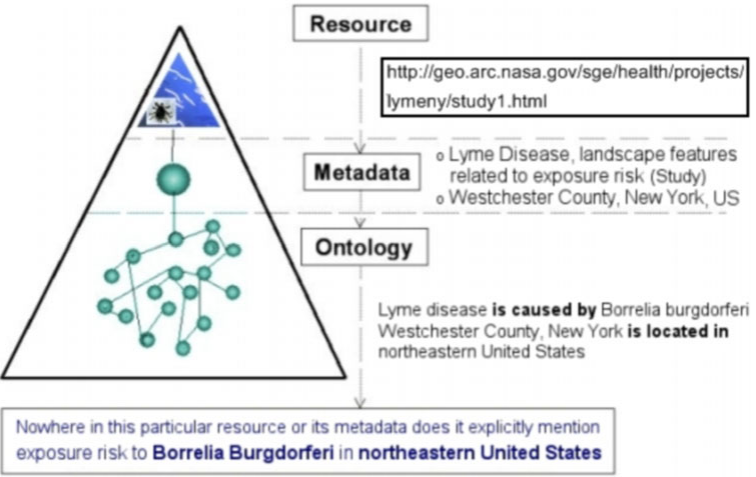
**Geo-ontology-aided semantic search of Web resources (Kamel Boulos, 2002)**. Metadata alone are not enough for successful retrieval of the Web resource/page shown in this figure. In this example, even though the resource and its metadata do not mention 'exposure risk to *Borrelia Burgdorferi *in north-eastern United States', a multiple ontology (geo-ontology and disease ontology)-assisted search for 'exposure risk to *Borrelia Burgdorferi *in north-eastern United States' would be able to find the resource [34].

Given such an expressive geo-ontology, location-based inferencing may be performed when mashing up geo-data corresponding to different levels of granularity. For example, given the parent-child relationship, data corresponding to a city can be integrated with the state/province in which it is located. In addition to geo-data mashup, semantic mashup can occur between different types of ontologies, including geo-ontologies and bio-ontologies (e.g., Gene Ontology [42] and Sequence Ontology [43]), and others (Figure 5 – [34,35]).

While machine-readable/machine-understandable data are essential to semantic mashup, most current Web content is only human readable. To bridge the gap between human readability and machine readability, RDFa (RDF attributes) [44] has been proposed to incorporate Semantic Web methods (RDF) into Web pages (i.e., into HTML – the HyperText Markup Language). RDFa provides a set of HTML attributes to augment visual data with machine-readable contexts. In addition to RDFa, the GRDDL (Gleaning Resource Descriptions from Dialects of Languages) specification [45] introduces markup based on existing standards for declaring that an XML (eXtensible Markup Language) document includes data compatible with RDF and for linking to algorithms (typically represented in XSLT – eXtensible Stylesheet Language Transformations [46]).

### Mashup song #4: from Yahoo! Pipes to 3-D, avatar-inhabited geo-mashups

**Figure 6:**
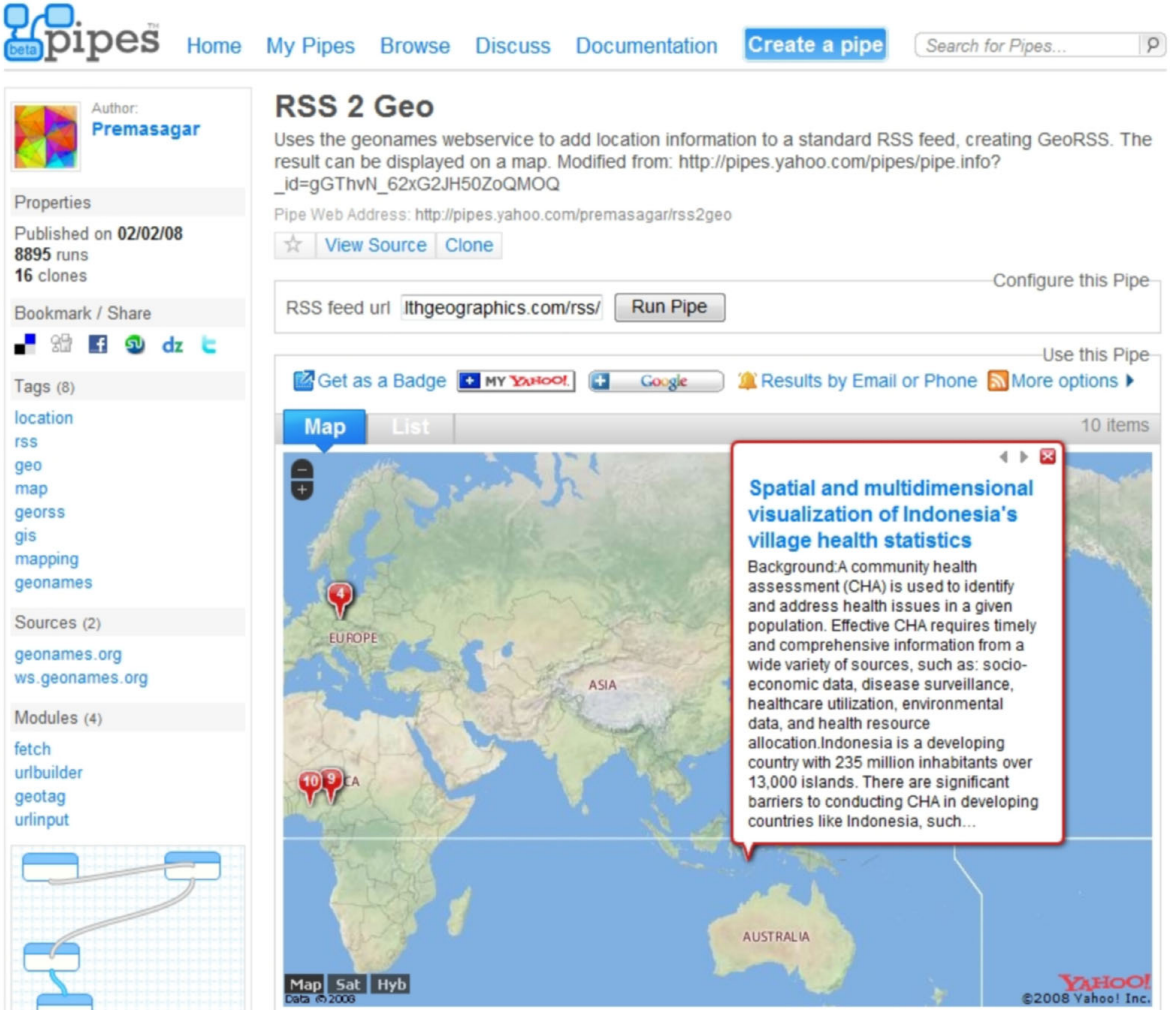
**Mapping the 'Latest articles' RSS feed from the *International Journal of Health Geographics *using the 'RSS 2 Geo' Pipe**. The screenshot shows one of the articles published by the *International Journal of Health Geographics *in June 2008 correctly mapped to Indonesia, the country name that appears in the article's title.

**Figure 7:**
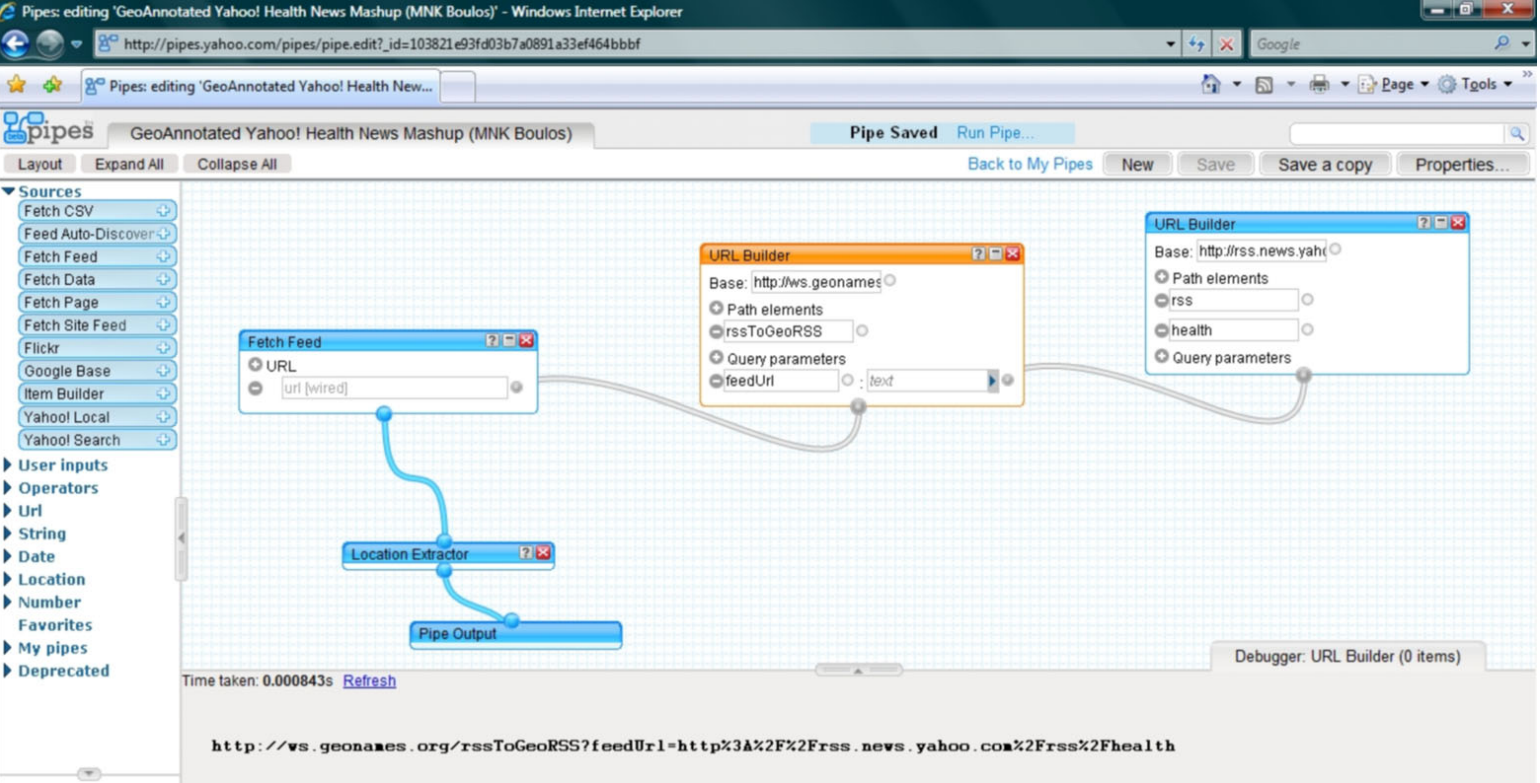
**Source of the 'GeoAnnotated Yahoo! Health News Mashup' in Yahoo! Pipes editor**. The URI of the GeoRSS output from GeoNames (the URL Builder module highlighted in orange in this screenshot) is shown in the gray pane at the bottom [54].

Like the first "song" in this "playlist", this "song" also starts in Yahoo! Pipes [22]. Yahoo! Pipes offers thousands of ready and free-to-use Pipes like the 'RSS 2 Geo' Pipe [47], which we were able to use to geo-encode and map the 'Latest articles' RSS feed from the *International Journal of Health Geographics *[48] as shown in Figure 6, without doing any coding or modification to the Pipe or to our RSS feed. Pipes can also be 'cloned', i.e., imported to one's account in Yahoo! Pipes, and then edited or remixed (as sub-pipes) in an intuitive, Web-based visual Pipe editor (Figures 2 and 7), to create and publish modified or more complex Pipes and mashups [49,50].

We cloned two publicly available, community-user-contributed Pipes originally created to map the latest news stories from Yahoo! and Reuters, with the goal of editing them to make them more specialized, so that they only map the latest *health *news items from Yahoo! and Reuters. The modified Pipes are available at [51]. They use the GeoNames RSS-to-GeoRSS Web service [39] to add location information to Yahoo! and Reuters' 'health' RSS newsfeeds (Figure 7). The results are then displayed using the Yahoo! Maps AJAX (Asynchronous JavaScript and XML) API [52], thanks to Yahoo! Pipes' Location Extractor module [53].

**Figure 8:**
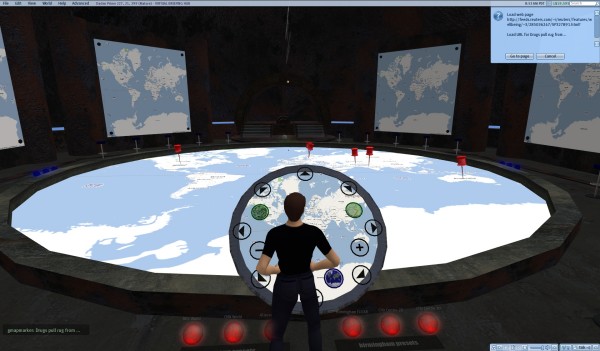
**'Reuters Features – Health & Fitness' GeoRSS feed displayed on Daden's "port" of Google Maps into the 3-D virtual world of Second Life**. The red pins on the map are mapped news items. Clicking any of them will prompt the user to display the corresponding news item in a Web browser. Our other 'Yahoo! News: Health News' GeoRSS feed looks and behaves the same when displayed on Daden's version of Google Maps in SL.

The Location Extractor module processes the GeoRSS output from GeoNames to map it in 2-D using Yahoo! Maps. The GeoRSS output from GeoNames is a URI [54], as can be seen in Figure 7. This URI can also be used outside Yahoo! Pipes, e.g., to map the GeoRSS feed on Google Maps instead of Yahoo! Maps [55], so we thought of feeding it into a "port" (by Daden Ltd [56]) of Google Maps in the 3-D virtual world of Second Life (SL) [57]. Figure 8 shows how the final output looks in Second Life. The remaining part of this geo-mashup "song" will provide some details about Daden's "port" of Google Maps into Second Life.

In March 2008, Linden Lab released a new version of Second Life, which for the first time let users display a Web page on the side of an object (or 'prim' as it is called in SL) within the world [58]. This was done using the same media channel that SL currently uses to display images and videos, and so was still restricted to one "page" per parcel, but it was at least a step forward. (A 'parcel' here refers to a circumscribed plot of virtual land in SL, with its own owner-customisable characteristics and settings.) However, the implementation does have two significant drawbacks:

• The page is not interactive, i.e., you cannot click on links in it; and

• You cannot scroll down or across the page.

Given our earlier work with maps in SL [2], we were interested to see how effective this new feature would be with Google Maps. Placing the URI of any Web page showing Google Maps rendered well within Second Life, but one could not zoom or pan on the map since the page was not interactive.

**Figure 9:**
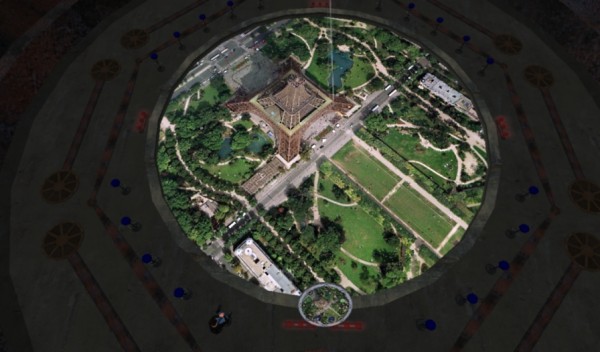
**Google Maps in Second Life**. In this snapshot, the 'Satellite' mode of Google Maps is displayed. The controller functions are implemented in the smaller circular panel, which appears near the centre-bottom of this screenshot (see also Figure 8).

In order to achieve this interactivity we built a small 'controller' within Second Life, and a simple Google Maps page generator on the Web. When first touched the controller sets the parcel URI to the URI of the page generator. The page generator, (effectively being called by SL to produce a Web page), generates a plain Web page consisting of a default whole-Earth map, and this is then rendered by the new SL Web page functionality on an object (a 10 m × 10 m square prim was used initially, but we also successfully used a 20 m diameter mega-prim). The controller sets variables for a default latitude/longitude of 0 degrees, and a default zoom of 15 (the further out that Google Maps goes). If the user then presses the 'zoom in' button on the controller (*not *on the Google Map), then the controller changes the CGI (Common Gateway Interface) parameter list on the URI to the new zoom value, which the generator script then uses with the Google Maps API to create a new zoomed-in page. In this way, all the standard Google Maps functionality of zoom in/out, pan and even 'Map', 'Satellite' and 'Hybrid' overlays was implemented in-world (Figure 9), with the controller tracking the state of the parameters and then sending them as CGI parameters to the page generator to create the relevant page.

The next challenge was how to represent data on Google Maps. Using our Newsglobe application [2], we could easily produce Google Maps with geocoded RSS or KML data overlaid as markers using the Google Maps API. However, although we could bring the map with markers image into SL using the process above, we could not then click on the markers to interrogate them (e.g., link out to the relevant news story or data reading). The solution was to bring the data itself into SL alongside the map (in a similar way to our Los Angeles aircraft visualisation described in [2]). Now, when a map with data is requested, the controller and page generator create the map in the standard way – with or without markers – but the controller also directly requests the data feed via a Web proxy which captures (and if necessary geocodes) the data from the RSS/KML feed and then passes them back into SL in a simple text format. The controller then uses these data to rez (SL term for 'resolve') a Second Life object (e.g., a map pin) at each location, and with each map pin hyperlinked back to the Web page containing the relevant item/story. If the user then zooms or pans the map, the controller de-rezzes the pins and then re-rezzes them in their new spatial position to reflect the zoom/pan, without having to re-request the data. A bounding box is applied to ensure that markers are not plotted well beyond the map. Given the 2048-byte limit on data coming in to SL, we typically also restrict the controller to bringing in only 10–20 data points at a time. We have however built in the ability to bring in multiple feeds, each feed being plotted in markers of a separate colour.

**Figure 10:**
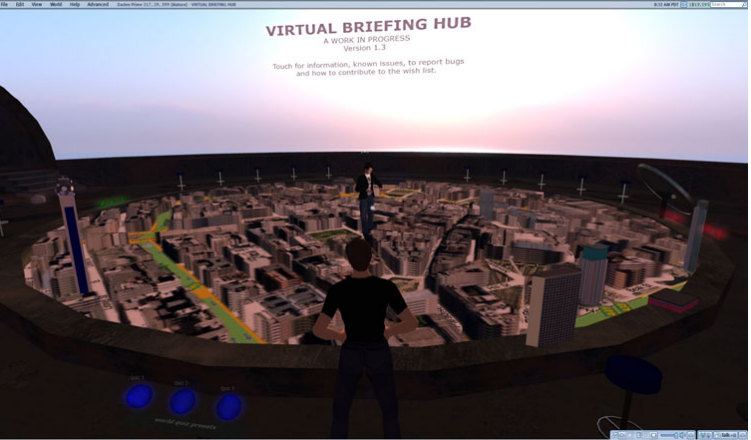
**Google Maps in Second Life**. The co-presence power of a 3-D virtual world: Besides feeling immersed in the data, the two humans/avatars in this snapshot can easily become aware of, and appreciate the presence of one another, get some sense of what the other can and cannot see, interact/chat while looking at the objects together, and even collaborate on modifying them and see the changes made by each other in real time.

**Figure 11:**
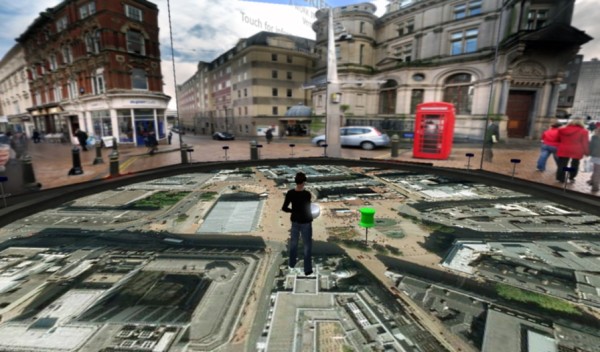
**Google Maps in Second Life**. Snapshot showing bespoke 360 degree panoramas keyed to particular locations on the map for an added sense of realism, immersiveness and of 'being there'.

Daden's first project for this system was with 'Digital Birmingham', the part of Birmingham City Council in the UK charged with promoting the use of digital technology within the city. They wanted Daden Ltd to create a 'Virtual Briefing Hub' (Figure 10), where they could explore the use of virtual world technology by planners and developers for regeneration, inward investment, education, infrastructure management, health and other fields. Using the above-mentioned Google Maps system in SL, it was relatively easy to zoom in on the city, augment the view with 2-D photos brought in by the Flickr API [59], news stories and Web sites from geocoded RSS feeds, and even some bespoke 360 degree panoramas keyed to particular locations on the map (Figure 11).

However, Daden also wanted to give a sense of the city in 3-D. They used two techniques to achieve this:

• For landmark buildings in the city (e.g., the BT Tower, Selfridges, Radisson SAS Hotel, Mailbox, Millennium Point) they created small scale models of each building and placed them on the right point of the map when zoomed in the central city area (Figure 10). The buildings match in horizontal and vertical scale at this zoom, but are disabled at other zooms.

• For the rest of the city they took the map image and used an image editor to make all the road and open spaces transparent. They then mapped this image onto a transparent object of the same size as the map in Second Life. They then stacked 5–7 layers of this on top of the map. The result was a pseudo-3-D effect, where the buildings show as if rising above the main map, but the open spaces are left at "ground" level (Figure 10).

For us this is just an initial step towards better 3-D mapping in Second Life – and better Web integration. Once Linden Lab release a full Web browser in Second Life (probably in 2009) then Daden's current approach will not necessarily be needed, although it does create a 3-D representation of the markers (pins), which no browser-only solution can easily achieve. It also certainly will not create the 3-D building models (without proper in-world support of specialist Web-browser plug-ins), so we think Daden's system will have significant longevity. Particular areas for enhancement, which Daden might wish to work on, include:

• Moving and scaling the 3-D objects as one zooms;

• Creating a separate and unique 3-D "layer" for each height "slice" within the city, enabling a truer representation of the city height profile to be obtained; and

• Importing Google Earth COLLADA models [60] for the individual 3-D buildings (COLLADA stands for COLLAborative Design Activity, an interchange file format for interactive 3-D applications).

## Discussion

### Other tools

In addition to the above-mentioned tools like Yahoo! Pipes [22] and Google Mapplets [9], which can be used for creating and publishing geo-mashups with little or no coding at all, there exist other equally effective ones worth exploring by interested readers to find out which tool (or combination of tools – or "instruments", to keep the musical metaphor going) works best for them and better serves their particular settings and purposes. For example, Google is now also providing Google Mashup Editor [61], an AJAX development framework and a set of tools that enable developers to quickly and easily create simple Web applications and mashups with Google services like Google Maps. Similarly, Microsoft has an interesting offering related to Yahoo! Pipes, which they call Microsoft Popfly [62,63].

**Figure 12:**
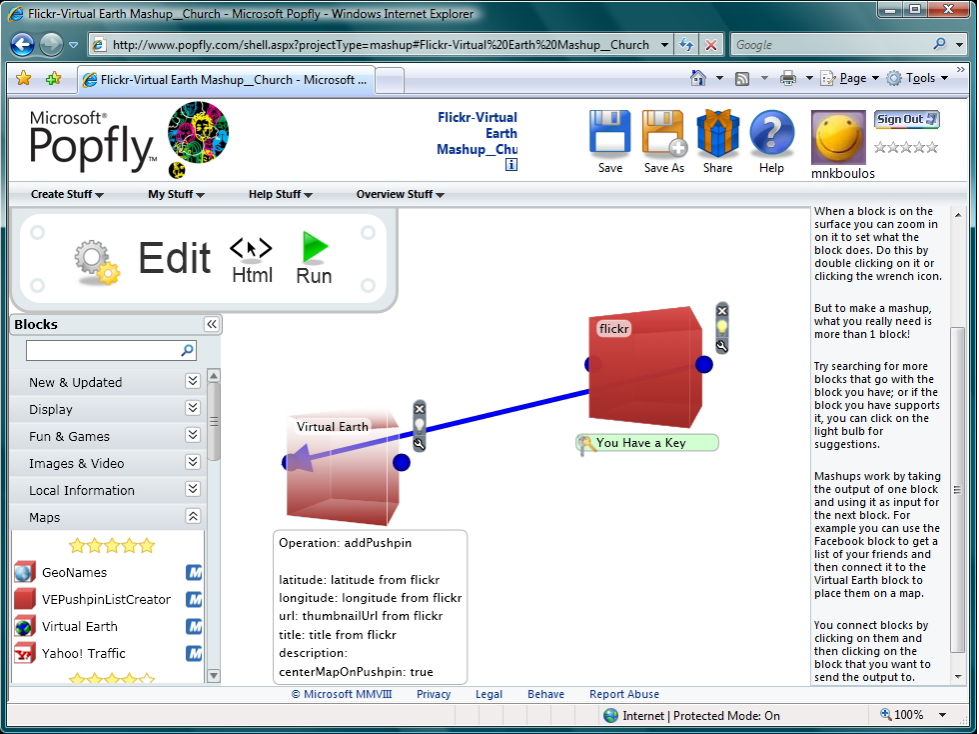
**The visual editor of Microsoft Popfly (using Microsoft Silverlight)**. This screenshot shows a very basic geo-mashup example using only two connected Blocks, Flickr [59] and Microsoft Virtual Earth, to search for and map geo-tagged Flickr images that match a given keyword. The output of this geo-mashup can be seen at [68].

Popfly allows users to create and publish Web pages, program snippets, and mashups using the Microsoft Silverlight rich Internet applications (RIAs) runtime [64,65] along with many pre-built 'Blocks' and data sources/services contributed by Microsoft and the user community, including a Virtual Earth Block for map display based on Microsoft Virtual Earth [66,67]. Blocks can be configured and visually linked to other Blocks in Popfly's intuitive Web-based editor, much like the visual "plumbing" in Yahoo! Pipes (Figure 12 – [68]). User-friendly error notices are given to users when incompatible data are passed between Blocks. There is also an advanced view for Blocks, which allows users to modify the underlying code in JavaScript, if they wish. Silverlight has been ported to Windows Mobile, and Nokia are to support it across a range of devices. Also, in a rare move, Microsoft announced Silverlight for Linux [69]. Adobe on the other hand is pushing its cross-platform AIR (Adobe Integrated Runtime) environment [70], which uses Adobe Flash, Adobe Flex, HTML, and AJAX to build RIAs.

### What's next: beyond Web 2.0 and the Semantic Web and towards the 3-D Internet and the ultimate Metaverse

The 'Google Maps/Earth in SL' tool described in "Mashup song #4" above, with its ability to visualize GeoRSS news and data feeds in the 3-D virtual world, is perhaps the first realization of the futuristic vision described by Wade Roush in [71]. However, Google Earth (the 3-D mirror world application) remains, to this date, far more powerful than its "port" in Second Life, like many other specialist data visualization tools, a fact echoed in a recent discussion of the topic by Paul Bourke [72]. For example, Google Earth has COLLADA support but not SL [2,60]. But despite this, there continues to be something very special or "magical" about the current avatar-inhabited Google Maps/Earth SL version by Daden (even with the medium's many current limitations)!

Miklos Sarvary, Director of the Centre for Learning Innovation at INSEAD, has drawn parallels between the life cycle of broadcasting and the Internet [73]: just as radio gave way to the more immersive experience of TV, today's flat Web sites will morph into more interactive, immersive multi-user experiences in which users can see and interact with each other in much more natural ways.

It is predicted that, within 5 to 7 years, the dominant Internet interface is likely to be the 3-D 'Metaverse', a new 3-D Web that will gradually "absorb", and seamlessly integrate with (not fully replace), today's World Wide Web and its applications like Google Earth [2,74]. (Today's 3-D virtual worlds are still rather early-stage technology and are yet to mature in order to fully realize this vision of a new 3-D Web.)

Today's flat Web allows us to call up "flat" information; a 3-D virtual environment allows us to more naturally experience and visualize this information in real-time with others, and also to appreciate their presence around us. Virtual worlds are such an appealing concept to users primarily because of the social 'co-presence' of others in these worlds in a very realistic manner (Figure 10).

When people are browsing the flat Web shop of Amazon.com, for example, they cannot see, chat with, and benefit from the experiences/opinions of, other people looking for the same items in real time, as they would do in a supermarket's aisle in the physical world. But with 3-D virtual worlds this is very possible.

Although there are some very early flat Web co-browsing solutions under development like Weblin ([75] – flat interface) and YOOWALK ([76] – two-and-a-half dimensional – 2.5-D interface) that have attempted to bridge this gap, they are not without their limitations, and it is expected that they will only achieve their full potential within 3-D online social/virtual worlds or the Metaverse over the coming few years. But this can only happen after the full 'HTML-on-a-prim' roadmap and vision [58], and many other currently missing or seriously lacking key features and qualities (e.g., better usability, scalability and cross-world interoperability) are properly developed and fully realized in these 3-D worlds.

In their introductory description of their MPK20 3-D Virtual Workplace [77], Sun Microsystems wrote under a paragraph entitled 'Why 3-D for Collaboration' at [78]: "*One question we are frequently asked is why use 3-D for a collaboration environment? While it might be possible to build a 2-D tool with functionality similar to MPK20, the spatial layout of the 3-D world coupled with the immersive audio provides strong cognitive cues that enhance collaboration. (...) In terms of data sharing, looking at objects together is a natural activity. With the 3-D spatial cues, each person can get an immediate sense of what the other collaborators can and cannot see*". (For other compelling arguments about the value of data visualization and collaboration in 3-D virtual worlds, please see [2,79,80].)

Humans are spatial beings by nature, inhabiting feature-rich 3-D analogue spaces, so a 3-D synthetic space should not be more cognitively demanding from a human-computer interface viewpoint compared to conventional flat interfaces, if it is properly designed with usability in mind. In fact, it could even make some presentations that are overly complex in 2-D version much less complicated to understand when ported to a more native 3-D environment.

Andrew Hudson-Smith and his team at the Centre for Advanced Spatial Analysis (CASA), University College London, have an extensive portfolio of GIS-related projects in Second Life, including: (i) Virtual London, (ii) a new approach to importing geographic terrains into Second Life as tabletops, and (iii) an Arc (ESRI) to Second Life project. In their popular 'Digital Urban' blog where these projects are described in detail [81], they frequently refer to Google Earth and Second Life as 'Three Dimensional Collaborative (Multi-User) Geographic Information Systems'. Second Life and Google Earth (and the related platforms that will definitely follow in the near future, as 3-D mirror and virtual worlds merge [2]) are indeed promising environments for public participation and collaboration type outreach activities, providing a good basis for a 'layered 3-D Wikipedia of the planet that anyone can edit and add to' or what can be referred to as 'The People's Atlas'. (Participatory GIS (PGIS) or Public Participation GIS (PPGIS) are terms that have been coined to express the adoption of GIS to broaden public involvement in policymaking, and thus empower local communities, especially the less privileged groups in society, who are often ignored in traditional government-oriented and run GIS applications.)

The 3-D Internet is also rapidly becoming a strategic European Commission (EC) research direction, with, for example, the recent establishment of three Working Groups (WGs) within a User Centric Media (UCM) cluster of 15 ongoing EC-funded projects in the area of Networked Media Systems: the Personalized & Creative Media WG, the 3D & Immersive Media WG, and the Future Media Internet WG [82].

However, we do appreciate that, for some (especially in the corporate domain), the public nature of a world like Second Life may be a barrier to adoption; despite the protection that one can put in place, the core data still goes through a third party server. However, IBM and Linden Lab are currently closely working on suitable solutions for this [83]. Moreover, recent months have seen significant developments in the OpenSim/Open Source SL-"compatible" platform and grids [84]. OpenSim lets users build worlds (and visualizations) on their own PC (Personal Computer) and servers, opened up only to the people they want and allow to access their worlds. In fact, one can now develop one's own spaces offline, publish them on any suitable offline digital storage medium, or host one's own live region/server on the Internet. These Open Source developments, coupled with recent work on an emerging 'MPEG-V for Virtual Worlds' ISO standard [85], can only lead to wider penetration of 3-D virtual worlds among online users and speed-up the development of interoperability specifications and protocols between these worlds [86].

Google's recent entry into the 3-D virtual worlds marketplace [87], as well as the availability of 3-D virtual worlds like Second Life on 3G (third generation) and WiFi enabled mobile phones and other small mobile devices, which is a reality today, thanks to an amazing and very well executed technology from Israel-based Vollee Ltd [88-90], and the novel and more natural 3-D world navigation devices and modalities that are emerging these days [91,92], will also serve to further expedite the mass penetration of 3-D virtual worlds among Internet users and the development of the next-generation 3-D Internet or Metaverse.

**Figure 13:**
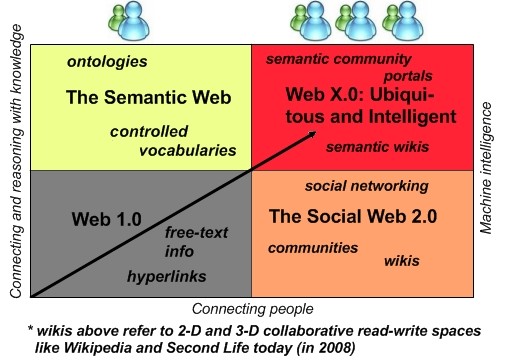
**Going beyond Web 1.0, Web 2.0 and the Semantic Web towards Web X.0 or the ultimate 3-D Metaverse**. Modified from an original diagram in [94] by Nova Spivak, Radar Networks; John Breslin, DERI; and Mills Davis, Project10X.

We believe that with the passage from the flat informational Web to the full 3-D experiential Web [82], which will ultimately happen over the next decade or so [93], a new Web will (have to) gradually emerge that combines the strengths of both the social Web 2.0 and the Semantic Web of today (Figure 13 – [1,94]), while also overcoming some of their respective weaknesses or deficiencies (e.g., the search and information retrieval problems in the social Web 2.0, with its "uncontrolled" or "loose" folksonomies and not uncommonly Lemmings-like 'wisdom of the crowds'; the apparent complexity, relative "restrictiveness" and "over formalism"/inaccessibility (from the viewpoint of ordinary end-users) of Semantic Web methods; and the increasingly non-textual nature of the Web, which presents many search/information retrieval and personalization challenges). These weaknesses or deficiencies can only become more problematic in a future, much more media-rich experiential Internet, if not properly addressed in the next-generation Web X.0 or Metaverse.

## Conclusion

The Web is still 'work-in-progress'. Nevertheless, over the past 5–7 years, Internet GIS has gradually transformed forever the way we approach and analyze geographic information, and has also changed the audience, both producers and consumers, of this information, making it today available to, and editable/remixable by, the wide masses, opening up the possibility of many new applications, and realizing the visions of community or 'Participatory GIS' and of the democratization or 'wikification' of GIS, or what has been called 'consumer health geoinformatics' [7].

Today, many online mapping applications exist where people can even add their own individual data to a shared Web map, e.g., 'Who is Sick?' [95,96]. And not just this, but also now users can dynamically overlay on similarly shared maps their own current position on Earth, and also view the position of others who have likewise shared their position, all in real-time over the Web, if they have, for example, a low-cost USB GPS (Universal Serial Bus Global Positioning System) mouse receiver or similar device connected to their PC or built into their mobile gadgets. GPS-enabled mobile phones and GPS-enabled cameras are enabling millions of people every day to collectively annotate the Earth in ways never done before, besides opening up many mobile location-based service possibilities and opportunities.

All of this is now possible and accessible like never before, thanks to the latest breed of 'neogeography' and 'GeoWeb 2.0' technologies and online services like Google Earth (a 3-D mirror world) and Yahoo! Pipes (a visual mashup creation and publishing service), but also not without its own newly introduced "problems" like copyright, individual privacy and even national security issues, all of which were not much the case when GIS was once very 'closed' and only the realm of big organizations and an elite of experts. We have previously discussed these side-effect issues and others in [2,7,21,74,79], but there is one more that seems very suitable for closing an article about geo-mashups.

The many easy-to-use online interfaces and visual mashup editors that are now available have increased the risks of wrong selection, manipulation and interpretation of data in some scenarios. As discussed in [97], the ideal consumer tools of the future need to be fault-tolerant and capable of analysing and presenting assembled data in ways that facilitate *only *appropriate interpretations of integrated or mashed-up data. This can be achieved by using some form of "intelligent", goal-oriented online health GIS wizards and mashup editors that are based on robust statistical, epidemiological and other methods, so that only valid results, maps and visualizations are allowed and produced, even when unlearned users attempt to select inappropriate settings or data for a particular analysis or geo-mashup.

## Competing interests

DB is Managing Director of Daden Limited, an Information 2.0 consultancy and full service Virtual Worlds/Second Life development agency. MNKB, MS, and KHC have no competing interests to declare.

## Authors' contributions

MNKB conceived and drafted the manuscript, and contributed parts of the Background, most of "Mashup song #4", all of the Discussion and Conclusions sections, as well as Figures 4, 5, 6, 7, 8, 10, 12 and 13. MS (with help from, and under KHC's supervision) contributed parts of the Background, Figures 1, 2, 3, and most of "Mashup songs #1-3", with the following exceptions: Figures 4 and 5, and some of the literature examples/pointers used in these sections, which were contributed by MNKB. DB contributed insider information/text (used in "Mashup song #4") about Daden's 'Google Maps in SL' tool, and also Figures 9 and 11. He also kindly allowed us to use the tool to render "Mashup song #4" in SL. All authors read and approved the final manuscript. Linden Lab, Second Life, SL, and SLurl are trademarks of Linden Research, Inc.

## References

[B1] Kamel Boulos MN, Wheeler S (2007). The emerging Web 2.0 social software: an enabling suite of sociable technologies in health and health care education. Health Information and Libraries Journal.

[B2] Kamel Boulos MN, Burden D (2007). Web GIS in practice V: 3-D interactive and real-time mapping in Second Life. International Journal of Health Geographics.

[B3] McFedries P (2007). The new geographers. IEEE Spectrum.

[B4] Mashup (web application hybrid) – Wikipedia. http://en.wikipedia.org/w/index.php?title=Mashup_%28web_application_hybrid%29&oldid=222992456.

[B5] Google maps. http://maps.google.com/.

[B6] Google earth. http://earth.google.com/.

[B7] Kamel Boulos MN (2005). Web GIS in practice III: creating a simple interactive map of England's strategic health Authorities using Google maps API, Google earth KML, and MSN virtual earth map control. International Journal of Health Geographics.

[B8] My maps – google maps user guide. http://local.google.com/support/bin/answer.py?hl=en&answer=68480.

[B9] Google Mapplets API. http://code.google.com/apis/maps/documentation/mapplets/.

[B10] Wood J, Dykes J, Slingsby A, Clarke K (2007). Interactive visual exploration of a large spatio-temporal dataset: reflections on a geovisualization mashup. IEEE Trans Vis Comput Graph.

[B11] Butler D (2006). Mashups mix data into global service. Nature.

[B12] HealthMap. http://www.healthmap.org/.

[B13] Google News. http://news.google.com/.

[B14] International society for infectious diseases, ProMED-mail. http://www.promedmail.org/.

[B15] World Health Organization (WHO). http://www.who.int/.

[B16] Google earth outreach – showcase public health. http://earth.google.com/outreach/p_health.html.

[B17] Turner A (2006). Introduction to Neogeography O'Reilly.

[B18] Show us a better way. http://www.showusabetterway.co.uk/call/.

[B19] O'Reilly T What is Web 2.0: Design patterns and business models for the next generation of software. http://www.oreillynet.com/pub/a/oreilly/tim/news/2005/09/30/what-is-web-20.html.

[B20] Dapper. http://www.dapper.net/.

[B21] Cheung KH, Yip KY, Townsend JP, Scotch M HCLS 2.0/3.0: Health care and life sciences data mashup using Web 2.0/3.0. J Biomed Inform.

[B22] Yahoo! Pipes: Rewire the web. http://pipes.yahoo.com/pipes/.

[B23] Official google maps API blog: Introduction and yahoo! pipes. http://googlemapsapi.blogspot.com/2007/04/introduction-and-yahoo-pipes.html.

[B24] FortiusOne GeoCommons. http://www.geocommons.com/.

[B25] GeoIQ Javascript API documentation. http://geoiq.earthinfomark.com/docs/geoiq_api_doc.pdf.

[B26] USGS, CDC ArboNet. http://diseasemaps.usgs.gov/.

[B27] Zou L, Miller SN, Schmidtmann ET (2007). A GIS tool to estimate West Nile virus risk based on a degree-day model. Environ Monit Assess.

[B28] Allen J (1976). A modified sine wave method for calculating degree days. Environmental Entomology.

[B29] NOAA NCDC: National climate data center. http://www.ncdc.noaa.gov/oa/ncdc.html.

[B30] CIPRES TreeBASE II: experimental google earth phylogenetic tree server. http://www.treebase.org/getrees/.

[B31] Janies D, Hill AW, Guralnick R, Habib F, Waltari E, Wheeler WC (2007). Genomic analysis and geographic visualization of the spread of avian influenza (H5N1). Syst Biol.

[B32] Mesquite Project: A modular system for evolutionary analysis. http://mesquiteproject.org/mesquite/mesquite.html.

[B33] Semantic web activity statement. http://www.w3.org/2001/sw/Activity.html.

[B34] Kamel Boulos MN, Roudsari AV, Carson ER (2002). Towards a semantic medical Web: HealthCyberMap's tool for building an RDF metadata base of health information resources based on the Qualified Dublin Core Metadata Set. Medical Science Monitor.

[B35] Kamel Boulos MN (2004). A first look at HealthCyberMap medical semantic subject search engine. Technology and Health Care.

[B36] W3C Semantic web activity. http://www.w3.org/2001/sw/.

[B37] OWL web ontology language overview. http://www.w3.org/TR/owl-features/.

[B38] GeoNames ontology – Geo semantic Web. http://www.geonames.org/ontology/.

[B39] GeoNames. http://www.geonames.org/.

[B40] Embrun, France. http://sws.geonames.org/3020251/.

[B41] Embrun, France (RDF). http://sws.geonames.org/3020251/about.rdf.

[B42] Ashburner M, Ball CA, Blake JA, Botstein D, Butler H, Cherry JM, Davis AP, Dolinski K, Dwight SS, Eppig JT, Harris MA, Hill DP, Issel-Tarver L, Kasarskis A, Lewis S, Matese JC, Richardson JE, Ringwald M, Rubin GM, Sherlock G (2000). Gene Ontology: tool for the unification of biology. Nature Genetics.

[B43] Eilbeck K, Lewis SE, Mungall CJ, Yandell M, Stein L, Durbin R, Ashburner M (2005). The Sequence Ontology: a tool for the unification of genome annotations. Genome Biol.

[B44] RDFa Primer: Bridging the human and data webs – W3C working draft 20 June 2008. http://www.w3.org/TR/xhtml-rdfa-primer/.

[B45] Gleaning resource descriptions from dialects of languages (GRDDL). http://www.w3.org/2004/01/rdxh/spec.

[B46] XSL Transformations (XSLT) version 1.0 – W3C recommendation 16 November 1999. http://www.w3.org/TR/xslt.

[B47] Pipes: RSS 2 Geo. http://pipes.yahoo.com/premasagar/rss2geo.

[B48] International journal of health geographics – latest articles (RSS feed). http://www.ij-healthgeographics.com/rss/.

[B49] Loton T (2008). Working with Yahoo! Pipes, No Programming Required.

[B50] Pruett M (2007). Yahoo! Pipes O'Reilly Safari Books Online.

[B51] GeoAnnotated health news mashups. http://pipes.yahoo.com/mnk_boulos.

[B52] Yahoo! maps ajax web services. http://developer.yahoo.com/maps/ajax/.

[B53] Yahoo! Pipes – operator modules: Location extractor module. http://pipes.yahoo.com/pipes/docs?doc=operators#LocationExtractor.

[B54] URI of the GeoRSS output from GeoNames for 'Yahoo! News: Health News'. http://ws.geonames.org/rssToGeoRSS?feedUrl=http%3A%2F%2Frss.news.yahoo.com%2Frss%2Fhealth.

[B55] 'Yahoo! News: Health news' GeoRSS feed displayed on Google maps using ACME GeoRSS Map viewer. http://www.acme.com/GeoRSS/?xmlsrc=http%3A%2F%2Fws.geonames.org%2FrssToGeoRSS%3FfeedUrl%3Dhttp%253A%252F%252Frss.news.yahoo.com%252Frss%252Fhealth.

[B56] Daden Limited. http://www.daden.co.uk/.

[B57] Daden brings Google Maps into second life (Second Life location SLurl). http://slurl.com/secondlife/Daden%20Prime/228/19/400.

[B58] Reuters/Second Life ≫ The last big feature "HTML on a prim". http://secondlife.reuters.com/stories/2008/03/27/the-last-big-feature-html-on-a-prim/.

[B59] Flickr services. http://www.flickr.com/services/api/.

[B60] Google Earth – Collada. http://www.collada.org/mediawiki/index.php/Google_Earth.

[B61] Google Mashup editor. http://code.google.com/gme/.

[B62] Microsoft popfly. http://www.popfly.com/.

[B63] Griffin E (2008). Foundations of Popfly: Rapid Mashup Development.

[B64] The Official microsoft silverlight site. http://silverlight.net/.

[B65] Map example from Silverlight Showcase. http://silverlight.net/world/.

[B66] Microsoft Virtual Earth. http://www.microsoft.com/VirtualEarth/.

[B67] Silverlight example using Microsoft Virtual Earth. http://silverlight.idvsolutions.com/.

[B68] Popfly example using Flickr and Microsoft virtual earth blocks. http://www.popfly.com/users/mnkboulos/Flickr-Virtual%20Earth%20Mashup__Church.

[B69] Microsoft Delivers Silverlight 1.0, Extends Support to Linux. http://www.microsoft.com/presspass/press/2007/sep07/09-04SilverlightPR.mspx.

[B70] Adobe strikes back at Silverlight with RIA. http://www.telecomseurope.net/article.php?id_article=5834.

[B71] Roush W (2007). Second Earth: The World Wide Web will soon be absorbed into the World Wide Sim: an environment combining elements of Second Life and Google Earth. MIT Technology Review.

[B72] Bourke PD Evaluating Second Life as a tool for collaborative scientific visualisation. Proceedings of Computer Games & Allied Technology 08: International Conference & Symposium on Computer Games, Animation, Multimedia, Security, IPTV & Edutainment: 28–30 April 2008; Singapore.

[B73] Sarvary M (2008). Breakthrough ideas for 2008: The metaverse – TV of the future?. Harvard Business Review.

[B74] Kamel Boulos MN, Hetherington L, Wheeler S (2007). Second Life: an overview of the potential of 3-D virtual worlds in medical and health education. Health Information and Libraries Journal.

[B75] Weblin. http://www.weblin.com/.

[B76] YOOWALK. http://www.yoowalk.com/.

[B77] MPK20 (video). http://www.youtube.com/watch?v=BJS8DGjeGvM.

[B78] MPK20: Sun's 3-D Virtual Workplace. http://research.sun.com/projects/mc/mpk20.html.

[B79] Huang ST, Kamel Boulos MN, Dellavalle RP (2008). Scientific Discourse 2.0. Will Your Next Poster Session Be in Second Life ^®^?. EMBO Reports.

[B80] McConaghy T Using second life for knowledge transfer and collaboration. Presented at International Workshop on Managing Knowledge for Space Missions: 17–19 July 2007; CalTech, Pasadena, California, USA.

[B81] Digital urban. http://digitalurban.blogspot.com/.

[B82] UCM cluster research input for the future internet research programme: Research on future media internet in a global context (March 2008). ftp://ftp.cordis.europa.eu/pub/fp7/ict/docs/netmedia/UCM-Position-paper.pdf.

[B83] IBM and Linden lab to explore enterprise-class solution for virtual world creation and collaboration (3 April 2008). http://www.businesswire.com/portal/site/google/?ndmViewId=news_view&newsId=20080402006512&newsLang=en.

[B84] OpenSim. http://www.opensimulator.org/.

[B85] International organization for standardization (ISO)/metaverse1 consortium: Draft requirements for the 'MPEG-V for 3-D virtual worlds' emerging standard (May 2008). http://www.virtualworldsnews.com/files/w9902_draft_requirements_for_mpegv.doc.

[B86] Linden Lab publishes draft Open Grid Protocol. http://wiki.secondlife.com/wiki/SLGOGP_Draft_1.

[B87] Lively by Google. http://www.lively.com/html/landing.html.

[B88] Vollee mobile access to world of second life. http://www.vollee.com/secondlife.

[B89] YouTube – second life streaming to your mobile phone by vollee. http://www.youtube.com/watch?v=XwRnjbkljnc.

[B90] YouTube – Vollee SL Client. http://www.youtube.com/watch?v=0Z2kXAbTn2c.

[B91] 3Dconnexion 3D mouse navigation enhances Second Life experience. http://www.3dconnexion.com/solutions/secondlife.php.

[B92] Handsfree 3D. http://www.handsfree3d.com/.

[B93] Virtual Worlds – A Roadmap to the Future?. http://www.daden.co.uk/downloads/Virtual%20Worlds%20-%20A%20Road%20Map.pdf.

[B94] Project10X's Semantic Wave 2008 Report: Industry Roadmap to Web 3.0 & Multibillion Dollar Market Opportunities. http://www.isoco.com/pdf/Semantic_Wave_2008-Executive_summary.pdf.

[B95] Who is Sick?. http://whoissick.org/sickness/.

[B96] Clark J (2008). The New Cartographers. What does it mean to map everything all the time?. In These Times.

[B97] Kamel Boulos MN (2005). Research protocol: EB-GIS4HEALTH UK – foundation evidence base and ontology-based framework of modular, reusable models for UK/NHS health and healthcare GIS applications. International Journal of Health Geographics.

